# The Impact of Normalization Methods on RNA-Seq Data Analysis

**DOI:** 10.1155/2015/621690

**Published:** 2015-06-15

**Authors:** J. Zyprych-Walczak, A. Szabelska, L. Handschuh, K. Górczak, K. Klamecka, M. Figlerowicz, I. Siatkowski

**Affiliations:** ^1^Department of Mathematical and Statistical Methods, Poznan University of Life Sciences, 60-637 Poznan, Poland; ^2^Institute of Bioorganic Chemistry, Polish Academy of Sciences, 61-704 Poznan, Poland; ^3^Department of Hematology and Bone Marrow Transplantation, Poznan University of Medical Sciences, 60-569 Poznan, Poland

## Abstract

High-throughput sequencing technologies, such as the Illumina Hi-seq, are powerful new tools for investigating a wide range of biological and medical problems. Massive and complex data sets produced by the sequencers create a need for development of statistical and computational methods that can tackle the analysis and management of data. The data normalization is one of the most crucial steps of data processing and this process must be carefully considered as it has a profound effect on the results of the analysis. In this work, we focus on a comprehensive comparison of five normalization methods related to sequencing depth, widely used for transcriptome sequencing (RNA-seq) data, and their impact on the results of gene expression analysis. Based on this study, we suggest a universal workflow that can be applied for the selection of the optimal normalization procedure for any particular data set. The described workflow includes calculation of the bias and variance values for the control genes, sensitivity and specificity of the methods, and classification errors as well as generation of the diagnostic plots. Combining the above information facilitates the selection of the most appropriate normalization method for the studied data sets and determines which methods can be used interchangeably.

## 1. Introduction

In recent years, RNA sequencing (RNA-seq) technology has become a mainstay of biomedical research and is an attractive alternative to microarrays. RNA-seq technologies have several advantages over microarrays, including less noise (the technical biases inherent in microarray technology are not present in RNA-seq experiments) [1], the possibility of detecting alternative splicing isoforms [2, 3], and the power to detect novel genes, gene promoters, isoforms, and allele-specific expression [4]. The decreasing cost of next-generation sequencing (NGS) is an additional argument for the selection of RNA-seq instead of microarray-based gene expression analysis. Despite the lower bias in RNA-seq experiment, there are still some sources of systematic variation that should be eliminated from RNA-seq data before the differential expression (DE) analysis. In particular, these variations include between-sample differences such as library size (sequencing depth) [5] or within-sample differences, for example, in gene length [6], guanine-cytosine (GC) content [7, 8], or unwanted variation introduced by batch effect [9]. Experience with microarray data has repeatedly shown that normalization aims to ensure that expression estimates are more comparable across features (genes) and samples. However, there are still a lot of questions about the impact of the normalization method on the results of RNA-seq data analysis. The significance of RNA-seq data normalization was demonstrated in [10]. Their main finding was that the choice of normalization procedure affects the results of DE analysis: sensitivity varies more between normalization procedures than between test statistics. Authors of [10, 11] showed that the normalization step raises questions, and there is still a need to provide useful practical tips and construct clear guidelines for researchers who may be unsure about how to choose a normalization method. To this end, we propose to apply a combination of graphical and statistical methods to compare the impact of the particular normalization on the results of DE analysis. Our investigation concerns five normalization methods widely used for normalization of RNA-seq data: Trimmed Mean of *M*-values, Upper Quartile, Median, Quantile, and PoissonSeq normalization implemented in R packages edgeR (v3.2.4), DESeq (v1.12.1), EBSeq (v1.3.1), and PoissonSeq 3 (v1.1.2), respectively. The comparison was based on the analysis of three data sets. Two of them are publicly available data sets (Bodymap and Cheung data) and one is the data set (AML data) coming from one of our projects (not published yet). In this paper, we outline a simple and effective method for comparing different normalization approaches and we show how researchers can improve the results of differential expression analysis by including in the normalization step different aspects based on the biology or informatics.

## 2. Materials and Methods

In this section, we describe the normalization methods to be compared and the data sets used in our study. Next, we propose the criteria for comparison of the impact of the normalization methods on the results of DE analysis.

### 2.1. Normalization Methods

Since the emergence of RNA-seq technology, a number of normalization methods have been developed. In our work we mainly focus on a comparison of five of the most popular normalization methods used for DE analysis of RNA-seq data, implemented in four Bioconductor packages: Trimmed Mean of *M*-values (TMM) [11] and Upper Quartile (UQ) [10], both implemented in the edgeR Bioconductor package [12], Median (DES) implemented in the DESeq Bioconductor package [13], Quantile (EBS) [10] implemented in the EBSeq Bioconductor package [14], and PoissonSeq (PS) normalization implemented in the PoissonSeq package [15]. All packages are available from CRAN (http://cran.r-project.org/web/packages) and Bioconductor (http://www.bioconductor.org/packages/release/bioc).

Because the basic source of variations between samples is the difference in library size (RNA samples may be sequenced to different depths), each library is assigned a normalization factor. There are several ways to calculate a normalization factor. We consider five different methods described as below.

Let us assume that we have *G* genes and *m* samples. Let *K*_*gj*_ and *K*_*gr*_ denote read counts for gene *g* and samples *j* and *r*, respectively. The *d*_*j*_ is the scaling factor for the *j*th sample.

The first presented method calculates a Trimmed Mean of *M*-values between each pair of samples. This method was proposed by Robinson and Oshlack [11] and is based on the hypothesis that most genes are not differentially expressed. The authors defined a normalization factor for a studied sample with a reference sample as follows:

(1) $$\begin{array}{c} \log_{2}\left(\;d_{j}^{\text{TMM}}\;\right)=\frac{\sum_{g\in G^{\prime }}^{}w_{gj}M_{gj}}{\sum_{g\in G^{\prime }}^{}w_{gj}}, \end{array}$$

where *M*_*gj*_ = log_2_⁡((*K*_*gj*_/*N*_*j*_)/(*K*_*gr*_/*N*_*r*_)), *w*_*gj*_ = (*N*_*j*_ − *K*_*gj*_)/*N*_*j*_ *K*_*gj*_ + (*N*_*r*_ − *K*_*gr*_)/*N*_*r*_ *K*_*gr*_, *K*_*gj*_, *K*_*gr*_ > 0. *N*_*j*_, *N*_*r*_ are, respectively, the total number of reads for sample *j*th and reference sample *r*, and *G*′ represents the set of genes with not trimmed *M*_*g*_ and *A*_*g*_ (absolute expression levels) values (according to [11] trimmed weighted mean is the average after removing the upper and lower percentage of the data; *M*_*g*_ values are trimmed by 30% and *A*_*g*_ values are trimmed by 5%). According to the assumption that most genes are not DE, *d*_*r*_^TMM^ should be close to 1 and log_2_⁡(*d*_*r*_^TMM^) = 0.

The next method, elaborated in [10], is Upper Quartile (UQ) implemented in the edgeR package. Here, the scale factor is calculated from the 75th percentile of the counts for each library after removing transcripts, which are zero in all libraries. It has the following form:

(2) $$\begin{array}{c} d_{j}^{UQ}=UQ\left(\;\frac{K_{gj}}{\sum_{g=1}^{G}K_{gj}}\;\right), \end{array}$$

where UQ(*X*) is the upper quartile of sample *X*, which is *j*th sample with normalized counts and *K*_*gj*_ > 0.

Anders and Huber [13] suggested the Median normalization method implemented in the DESeq Bioconductor package. This method makes the same assumption as the TMM method (the majority of genes are not DE). A scaling factor for a given *j*th sample takes the median of the ratios of observed *j*th sample's counts to the geometric mean across samples (i.e., a pseudoreference sample):

(3) $$\begin{array}{c} d_{j}^{\text{MED}}={\text{median}}_{g}\frac{K_{gj}}{{\left(\prod_{v=1}^{m}K_{gv}\right)}^{1/m}}. \end{array}$$

It is also feasible to perform Quantile normalization across samples, as is often done in the case of microarray data [10]. Here, we used Quantile normalization that is implemented in the EBSeq Bioconductor package [14]. This normalization method estimates the sequencing depth of an experiment by an upper quartile of its counts and has the following form:

(4) $$\begin{array}{c} d_{j}^{Q}=10^{{log}_{10}Q_{j}-\left(1/m\right)\sum_{v=1}^{m}{log}_{10}Q_{v}}, \end{array}$$

where *Q*_*j*_, *Q*_*v*_ are the upper quartiles of the *j*th sample and *v*th sample, respectively. This method aligns the distribution of all samples.

Finally, we also tested a normalization method (PS) proposed in [15], implemented in the PoissonSeq package. It has the following formula:

(5) $$\begin{array}{c} d_{j}^{PS}=\frac{\sum_{g\in G^{\prime \prime }}^{}K_{gj}}{\sum_{g\in G^{\prime \prime }}^{}\sum_{j=1}^{m}K_{gj}}, \end{array}$$

where *G*′′ is the set of genes found by goodness-of-fit statistics of the form:

(6) $$\begin{array}{c} {\text{GOF}}_{g}=\overset{m}{\underset{j=1}{\sum }}\frac{{\left(K_{gj}-d_{j}^{TC}\cdot \sum_{j=1}^{m}K_{gj}\right)}^{2}}{d_{j}^{TC}\cdot \sum_{j=1}^{m}K_{gj}}, \end{array}$$

where

(7) $$\begin{array}{c} d_{j}^{TC}=\frac{\sum_{g=1}^{G}K_{gj}}{\sum_{g=1}^{G}\sum_{j=1}^{m}K_{gj}} \end{array}$$

is the Total Count (TC) scaling factor. We chose genes to the set *G*′′ for which the values of GOF_*g*_ are in interval (0.25; 0.75).

Furthermore, we compared the performance of all normalization methods mentioned above against unnormalized data, denoted by “raw data” (RD).

In addition, another source of variation, related to GC-content and batch effect, was also considered. Obtained results revealed that inclusion of GC content analysis did not contribute to the proposed strategy of comparison and did not influence the summary ranking (see Figure S5 and Table S4 in Supplementary Material available online at http://dx.doi.org/10.1155/2015/621690). Therefore, this normalization strategy was not included in further analysis.

The undesirable variation coming from batch effects, such as sampling time, different technology, can be adjusted with the help of methods implemented in sva package [9]. We only had information about the batches in the AML data that could be introduced by two sampling dates. That is why we only considered such approach for AML data. Before including additional normalization we checked whether the presence of batch effects in AML data sets existed. Detecting the presence of batch effects in AML data was accomplished in two ways. We have provided a hierarchical clustering (see Figure S8) together with principal component analysis (see Figure S9). Our results did not confirm existence of batch effect connected with sampling date in the case of AML data. Therefore, this normalization strategy was neglected in further analysis.

### 2.2. Data Sources

The five normalization methods were compared based on the analysis of three real RNA-seq data sets. Two data sets included in this study were obtained from publicly available resources (Bodymap and Cheung data) and one was obtained from the project performed at the Institute of Bioorganic Chemistry in Poznan (AML data). The Bodymap data set was published in [16], where the transcriptome of nontransformed human mammary epithelial cells in reference to Illumina Bodymap data collected from normal tissues was studied. The Cheung data set [17] comes from the study of gene expression of human B cells from individuals belonging to large families. The aim of the study was the identification of polymorphic transregulators. Both of these data sets are deposited in the Recount database at http://bowtie-bio.sourceforge.net/recount/ [18].

The AML data set comes from our as yet unpublished RNA-seq experiment. We studied gene expression profiles in 30 peripheral blood (PB) and bone marrow (BM) samples obtained from 25 adult AML (acute myeloid leukaemia) patients cured at the Department of Haematology and Bone Marrow Transplantation, Poznan University of Medical Sciences. Two samples per lane were sequenced at the Institute of Bioorganic Chemistry in Poznan, using single-read flow-cell (SR-FC) and Genome Analyzer IIx (GAIIx, Illumina). As a control, one BM sample and a pool of 12 PB samples obtained from healthy volunteers were sequenced on one additional lane. The libraries were prepared from up to 4 *μ*g total RNA with the use of the TruSeq RNA Sample Preparation Kit v2 (Illumina), according to the instructions of the manufacturer, and validated with a DNA 1000 Chip (Agilent). Each library generated approximately 20 million 50-nt-long reads, processed in CASAVA, FastQC, and the NGS QC Toolkit. The reads were mapped to the reference human genome UCSC hg19 with TopHat run (v2.0.6).

Before all calculations we filtered the data sets to obtain a similar amount of genes in each data set. From the Cheung and Bodymap data sets we chose only those genes for which the mean of the counts for all samples was greater than 0, whereas in the AML data set we chose 50 as the cut-off value for the mean of the counts in all samples. The summarised information about each data set is presented in Table 1, while the details can be found in Supplementary Table S1 and Figure S1. As we can see, the data sets varied with the sample sizes and gene numbers as well as the gene expression levels. In Cheung data set, genes with low levels of expression predominate. In Bodymap data set, the biggest group is constituted by the genes with high levels of expression, while in the AML data set the biggest group is constituted by the genes with medium levels of expression. Such variety of data sets enables revealing differences of performance of normalization methods in case of data with different structure of genes.

### 2.3. Analytical Criteria for Comparison of Normalization Methods

#### 2.3.1. Selection of the Housekeeping Genes (HG)

The idea of using HG arose from the previous research concerning microarray experiment. In that experiment dye-swapped microarrays were used. Chosen dyes played the role of housekeeping genes. In considered RNA-seq experiments we do not have the lists of housekeeping genes straightforwardly. That is why we decided to perform our investigation of the impact of normalization methods based on analytical version of HG lists, genes similarly expressed across samples.

The square root of the mean square error was used as a measure. The housekeeping genes were selected based on the raw data by using the following formula:

(8) $$\begin{array}{c} {MSE}_{g}=\sqrt{\frac{\sum_{j=1}^{m}{\left(K_{gj}-{\overset{-}{K}}_{g\cdot }\right)}^{2}}{m}}, \end{array}$$

where ${\overset{-}{K}}_{g\cdot }$ is the mean of the counts for gene *g*. We decided to choose housekeeping genes for all data sets based on a particular cut-off value and applied a linear transformation of the results to the interval [0,1] using min-max normalization:

(9) $$\begin{array}{c} {\hat{MSE}}_{g}=\frac{{\text{MSE}}_{g}-\min\left({\text{MSE}}_{g}\right)}{\max\left({\text{MSE}}_{g}\right)-\min\left({\text{MSE}}_{g}\right)}. \end{array}$$

Then we selected 1% of all genes with the lowest $\hat{MSE}$ values as the housekeeping genes. Although the number of housekeeping genes was the same in each data set, different genes were included for each set. The tables and bar plots concerning housekeeping gene selection are provided in Supplementary Materials (Table S2 and Figure S2). These indicate the same facts as for the case with all genes taken into account. There is some variability in data sets with respect to number of HG genes for each abundance level of reads. In all data sets we can see that there is the highest number of HG genes with number of reads in genes below 500 (relative medium abundance level).

#### 2.3.2. Bias and Variance

To evaluate the normalization methods used for the processing of RNA-seq data, we applied the bias and variance criterion proposed by Argyropoulos et al. [19] for the analysis of double-channel microarray data. We adjusted earlier this method for one-channel microarray data [20]. In this paper, we transform the method to be suitable for the RNA-seq data. We can use the following formulae of bias and variance as proxies accuracy and precision, respectively:

(10) biasi=1m∑j=1mlog2KijK−i·−True Log Ratio2,

(11) variancei=1m−1∑j=1mlog2KijK−i·−log2KijK−i·¯i2,

where *K*_*ij*_ denotes read counts for *i*th control gene from the *j*th sample, ${\overset{-}{K}}_{i\cdot }$ is the mean counts for the *i*th control gene and $\bar{{log}_{2}\left(K_{ij}/{\overset{-}{K}}_{i\cdot }\right)}$ is the mean value of ${log}_{2}\left(K_{ij}/{\overset{-}{K}}_{i\cdot }\right)$ for the *i*th control gene, and True Log Ratio is the true value of ${log}_{2}\left(K_{ij}/{\overset{-}{K}}_{i\cdot }\right)$ for the *i*th control gene from the definition of HG. The accuracy of a measurement is the degree of closeness of measurements of a quantity to the true value. The precision of a measurement is the degree to which repeated measurements under unchanged conditions show the same results. Based on the definition, each gene, which is considered as HG, should have the same number of counts in each sample, as well as mean value of counts for this gene. Thus, the True Log Ratio is equal to 0 and the formula (10) for the bias reduces to the root mean square error (RMSE):

(12) $$\begin{array}{c} {\text{bias}}_{i}=\sqrt{\frac{1}{m}\overset{m}{\underset{j=1}{\sum }}{\left({log}_{2}\left(\frac{K_{ij}}{{\overset{-}{K}}_{i\cdot }}\right)\right)}^{2}}. \end{array}$$

The ratios (bias and variance) for each normalization method will be the mean of bias and variance values computed for all of the control (housekeeping) genes. The normalization method “A” would be preferred over the method “B” if it is associated with the smallest bias and variance [19].

#### 2.3.3. Differential Expression Analysis

Our goal was to find out how normalization methods affect differential expression results. Therefore, after application of each normalization method, differential expression analysis was performed using the edgeR method from the edgeR Bioconductor package [12]. This method was chosen because it showed reliable performance in wide range of experiments and it allows easy inclusion of scaling factors into the statistical test. The edgeR method was specifically developed to model count data dispersion and it is designed for overdispersed RNA-seq data. Briefly, it is assumed that counts are a negative binomial distribution, according to

(13) $$\begin{array}{c} K_{gj}\sim NB\left(\;d_{j}\lambda_{gj},\phi_{g}\;\right), \end{array}$$

where *λ*_*gj*_ is the expression level of gene *g* in sample *j*, *d*_*j*_ is the normalization factor in sample *j*, and *ϕ*_*g*_ is the dispersion of gene *g*. The method of differential expression analysis, implemented in the edgeR package, extends Fisher's exact test.

#### 2.3.4. Prediction Errors

Discriminant analysis was applied to determine the effectiveness of the sample classification based on found DEGs identified in each data set after each normalization procedure. Different classification methods may lead to different prediction errors. We estimated the classification errors based on the five classifiers: naive Bayes, neural network, *k*-nearest neighbour, and support vector machines and random forest that are presented in Table 2. Analyzing each data set, we used leave-one-out cross validation (LOOCV) to obtain the estimate of the predictor errors of the test set that results from using different classifiers. For a data set with *n* samples, this method involves *n* separate runs. For each run, a number of samples, minus one data point, are used to train the model and then prediction is performed on the remaining data point. The overall prediction error is the sum of the errors for all *n* runs [21]. As input for the classifiers in the discriminant analysis we chose these informative genes, having high power of discrimination that are differentially expressed. The set of genes was obtained by gene selection process from a test statistic after each normalization procedure.

All computations and diagnostic plots were performed with R 3.0.2 [22].

## 3. Results

The aim of our study was to compare various normalization approaches and outline the workflow that will help to select the normalization method appropriate for a particular data set. We decided to test five of the most commonly used methods of RNA-seq data normalization: Trimmed Mean of *M*-values (TMM), Upper Quartile (UQ), Median (DES), Quantile (EBS), and PoissonSeq (PS), described in detail in the Methods section. All methods were tested on three different data sets: Bodymap, Cheung, and AML data. The details concerning data sets are described in Section 2.

### 3.1. Differential Expression (DE) Analysis

The main purpose of many RNA-seq experiments is to identify genes that are differentially expressed between the compared conditions. Analyzing three mentioned above data sets, we checked the direct impact of the normalization methods described above on the results of DE analysis. After each normalization, we determined the lists of differentially expressed genes (DEGs) using the statistic test from the edgeR package. In the case of each data set, we compared gene expression levels between two types of biological samples and ranked the genes according to adjusted *P* values. Genes that had adjusted *P* values < 0.05 were selected as differentially expressed.

**Figure 1:**
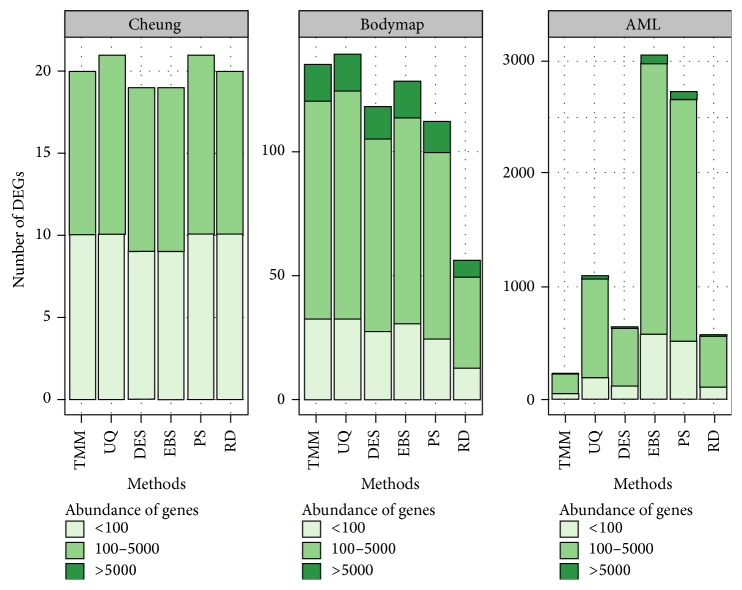
Bar plots of the DEGs with specified levels of count abundance in all studied data sets. On the *x*-axis the methods of normalization are featured, whereas the *y*-axis represents the number of DEGs determined after each normalization procedure. The bar colours represent the groups of genes of particular level of expression.

The results of DE analysis can be compared based on the number and content of DEGs. Visualisation of the DEGs is presented in Figure 1. The bar plots show the contribution of genes with particular expression levels in DEG lists for all data sets, selected from data submitted to five tested methods of normalization and raw data (RD). As we can see, the influence of normalization methods differs between data sets. In the case of the Cheung data set, all methods result in a similar number of DEGs and their significant part constitutes genes with low levels of expression. A more balanced contribution of DEGs was observed in the Bodymap data set; the number of DEGs with a middle expression level is slightly higher than the number of very highly or weakly expressed genes. In AML data set, genes with an average expression level clearly predominate on the list of DEGs. Here, there are also the most significant differences in the number of DEGs between variously normalized data. The most restrictive method seemed to be TMM, whereas the highest numbers of DEGs were obtained by EBS and PS methods. The contribution of all groups of genes is the same for each normalization method.

MA plots, available in Supplementary Materials (Figure S3), were generated for additional visualization of DEGs. They present the relationship between base mean and log2FC of the counts. The results show that DEGs in each MA plot after normalization are slightly more spread out compared to the raw data. However, the location of DEG differs depending on the normalization method for AML and Bodymap data sets. In the case of AML data it is also worth pointing out that we can observe more DEGs that are overexpressed than underexpressed.

As it is difficult to evaluate which normalization method is more suitable for a data set based on MA plot analysis, more precise verification is necessary. To this end we calculated bias and variance values.

### 3.2. Bias and Variance Calculation

Based on details described in the previous section we selected housekeeping genes for which we calculated bias and variance values. Following the idea described in the study [19], we assumed that the most appropriate normalization method is that which generates the lowest bias and variance values for the control genes. The bias and variance values were calculated according to formulae (11) and (12) for all the housekeeping genes selected separately for each data set, as described in Section 2. Then, for each data set, the mean of bias and variance values for each normalization method and for all control genes was calculated. Tables 3 and 4 present the ranking of normalization methods based on these bias and variance values; the method with the lowest bias or variance was ranked as 1. The highest bias and variance values, at least in the Cheung and Bodymap data sets, were observed for the unnormalized raw data (RD), included into the tables for comparison. Taking into account all of these data sets the conclusion is that the best results were obtained using DES, EBS, and PS normalization methods, and the methods which generated the highest bias and variance values were TMM and UQ. In the case of AML data, application of TMM method led to increase of the bias and the variance present in RD. It is worth pointing out that the differences between them are small in all data sets.

### 3.3. Sensitivity and Specificity

The sensitivity and the specificity of the normalization methods were investigated using the AML data set, based on our earlier experience with the analysis of microarray data, described in [20] as well as evidence from literature [23, 24]. First, the sets of genes were selected as positive and negative controls. The positive controls consisted of the genes that were strong candidates for DEGs. For the set of positive controls, we selected genes that were validated by real-time polymerase chain reaction (PCR) analysis or described in the literature as overexpressed (or, less frequently, underexpressed) in AML or immature hematopoietic cells [23]. The negative controls included the genes that are not DEGs (their expression levels should not differ between these two types of samples) [24]. In total, 44 genes were chosen as the positive controls and 44 genes were chosen as the negative controls. The lists of genes can be found in Table S3. The sensitivity and specificity of the normalization methods were calculated, respectively, as a percentage of positive controls that were present and the percentage of negative controls that were absent in each list of differentially expressed genes. The normalization methods with the highest values of specificity better show nondifferentially expressed genes, while on the other hand, the methods with the highest sensitivity values indicate a high probability of finding DEGs that are truly DEGs. The results of this analysis are presented in Table 5.

Table 5 shows that the specificity values for EBS and PS methods are substantially lower than for the remaining ones. The sensitivity values were less divergent between the methods and were generally low, in the range between 10 and 31%. Furthermore, for TMM and EBS methods we obtained the most divergent results: the highest specificity but the lowest sensitivity for TMM, and the opposite for EBS. In the case of specificity, we can see that most of the methods produce values of specificity over 80%.

### 3.4. Prediction Errors

Table 6 presents a comparison of the five normalization methods when for each data set the different numbers of informative genes were used based on five classifiers and LOOCV. In each case we selected the number of differentially expressed genes as 75% of the number of samples. Therefore for the Cheung, Bodymap, and AML data sets we have, respectively, 30 (0.75*∗*41), 12 (0.75*∗*16), and 20 (0.74*∗*27) differentially expressed genes. The results in the table suggest that, in all data sets, PS, DES, and EBS perform better than TMM and UQ.

### 3.5. Diagnostic Plots

**Figure 2:**
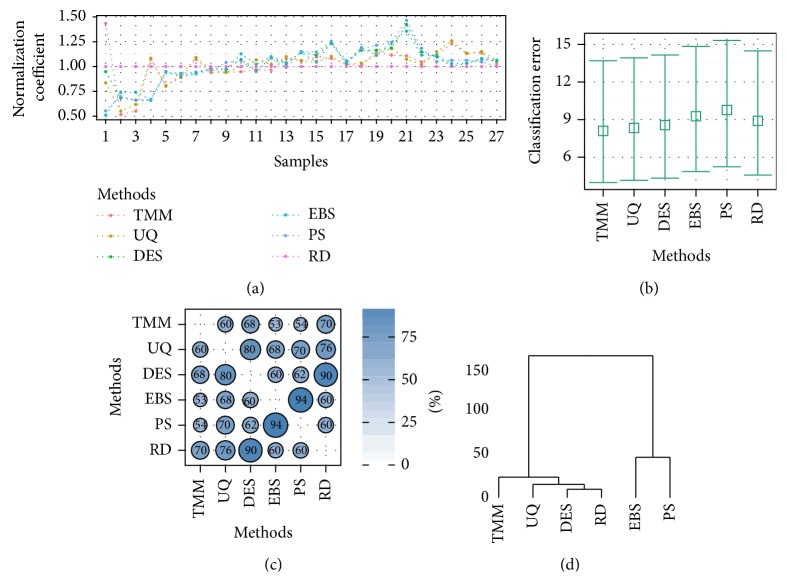
Diagnostic plots for the AML data set. Besides the five normalization methods, raw data (RD) were also included in the plots as a benchmark. (a) presents calculated normalization factor values across the samples by each method. The samples are ordered by the minimum values of normalization factors. (b) shows 95% confidence intervals for the mean of the percentages of classification errors calculated for each method based on five selected classifiers. (c) shows the numbers of common DE genes across each pair of normalization methods. The size and shading of the circles represent the average percentage value of common genes between each pair of methods. (d) presents the results of clustering of the normalization methods based on 20 common DE genes found by each of these methods. A dendrogram was created with hierarchical clustering based on Ward's method.

Besides the analytical methods for comparison of normalization methods we suggest using additional determinants that may be helpful for the rejection of the most commonly used methods that evidently fail. It is possible that the normalization methods yield different results for different data sets. Therefore, we suggest the application of the following workflow based on diagnostic plots to determine which normalization method is optimal for a specific data set. Here, we focus only on the AML data set. In Figure 2(a) we can observe the differences introduced to normalization factors obtained by each normalization method. From this figure we can conclude that normalization coefficients determined with TMM and UQ methods group together and divergent from the rest of normalization methods.

The results in Table 6 may be summarised considering the average errors expressed through figures. For the AML data set the performances of the five normalization methods can be ranked. The percentages of classification errors obtained by normalization methods could be used to obtain a 95% confidence interval of the mean of the proportion of classification errors for the normalization. The corresponding bar chart plotting confidence intervals is given in Figure 2(b). This plot indicate that the TMM, UQ, and DES methods outperform the two other methods with respect to classification performance.

### 3.6. Common DEGs

To compare the number of DE genes and the number of common DE genes found among the normalization methods performed for a particular data set, we generate balloon plots (Figure 2(c) and Figure S5) and Venn diagrams (see Figure S4). Balloon plots represent percentages of the number of commonly detected differentially expressed genes between the *i*th and *j*th methods. First we calculate in the (*i*, *j*)th cell a proportion of common detection with respect to the *i*th method:

(14) $$\begin{array}{c} P_{ij}=\frac{D_{ij}}{D_{i}}\cdot 100, \end{array}$$

where *i* ≠ *j*, *D*_*ij*_ is the number of differentially expressed genes commonly detected by the *i*th method and the *j*th method, and *D*_*i*_ is the number of differentially expressed genes detected by the *i*th method. Next, we take the average percentage value of common genes between each pair of methods. The most preferable method is the one with the highest number of common DEGs.

For the AML data set (Figure 2(c)) the lowest number of common DEGs with other methods is produced by the TMM normalization method, and the best by the EBS method. The lowest number of common DEGs was obtained for the EBS–TMM and PS–TMM comparisons. From this analysis we concluded that the set of genes identified as DEGs is not stable and depends on both the method of data normalization and the data set itself. However, in the case of AML data set, there is a set of 227 genes common for all tested methods (Supplementary Figure S4) and these genes can be considered as the strongest candidates for DEGs (Figure S4). In the case of the Cheung data set, we noted that most of the methods appear to perform similarly. For the Bodymap data set, all normalization methods yielded slightly lower percentages of commonly detected DEGs than in the case of the Cheung data set. For the raw data (RD), the number of common genes for any pair of methods was below 50% (see Figure S5). The Venn diagrams for the Cheung and Bodymap data sets also confirmed this tendency (Figure S4).

### 3.7. Grouping of Normalization Methods

Clustering is another approach to compare the performance of the normalization methods. Based on the similarities between the DE gene lists, we generated dendrograms to easily observe which methods group together. The exact measure of similarity was the DE gene rank. In the case of each data set, for five variants of normalized data, as well as for raw data (RD), particular sets of differentially expressed genes were obtained. First, we chose genes common to all six DEG lists. Since we obtained 20 genes in common between six methods, we performed the clustering of the normalization methods based on these common DEGs. Then, for all methods, we ranked these genes, thus obtaining ranking lists of genes. Based on these lists, we computed the distance matrix by using Euclidian distance and plot dendrograms (Figure 2(d)). The dendrograms were constructed from hierarchical clustering using Ward's method. This criterion is another approach that compares the DEGs lists. The previous criterion (common DEGs) gives us the information about the percentage of DEG in common per pair of methods, whereas this criterion gives the information about the similarity between the methods based on the order of common DEGs in each list.

### 3.8. Summary of Ranks

Combining all of the criteria described in the previous sections, we would like to determine which of the five tested normalization methods would be appropriate for the AML data set.  Table 7 summarises the ranks obtained based on the bias and variance values, the prediction errors, sensitivity, specificity, and the number of common DEGs for the AML data set. In the case when all the criteria included in Table 7 are equally important, the investigator can base the decision on the final rank calculated as the mean of the ranks established separately for each criterion using chosen normalization methods.

## 4. Discussion

In practice, normalization of high-throughput data still remains an important question and has received a lot of attention in the literature. The increasing number of normalization methods makes it difficult for scientists to decide which method should be used for which particular data set. Based on the results presented in this paper as well as in [25] we can conclude that normalization affects differential expression analysis; therefore, an important aspect is how to choose the most sensible method for the data. In this paper we have shown that, depending on the data structure, the influence of normalization differs (see Figures 1 and 2(d)). In our work we have demonstrated that based on some of these criteria the choice of normalization method can be more suitable and robust and can be made more automatically. Coefficients such as bias and variance can be considered as criteria for a comparison of normalization methods. It is worth pointing out that in our investigation in most cases including the normalization reduces the bias and variance values compared to raw data, which confirms the need of normalization. When the differences between the bias and variance values are significant, the usage of ranks of these values reflects the real differences more precisely. However, in our investigation the differences between obtained bias and variance values for all methods in all data sets are small. In such case, using ranks does not reflect the true differences between methods and additional criteria are needed. The diagnostic plots could serve as such additional determinants and may be helpful for the rejection of the most leading methods that evidently fail. Our study indicates that the use of TMM method in most cases is displayed poorly. This conclusion is not in agreement with the evaluation made by [26, 27]. Their study indicated that the use of TMM method led to good performance on simulated data sets. One reason for the disagreement of these results can be due to different approaches of comparison and usage of criteria not considered by other authors. Another reason of differences in the conclusions could arise from the number of biological replicates used in our study or could be related to the particular data sets used in these papers.

Furthermore, we find that other conclusions described in [26] are consistent with our own results. These results confirm the satisfactory results of the DESeq method. In Table 7 we presented the summary of ranks for the AML data set. It is possible that the normalization of other data sets can yield results that are different from those obtained with the AML data set (in this paper we have shown that, depending on the data structure, the influence of normalization differs). In general, each criterion proposed in the paper focuses on different aspects of comparison. Depending on the main objectives of the research some of the criteria could be more useful; for example, if impact lies in good prediction based on chosen genes the important aspect will be prediction errors. However in some cases the results of criteria are inconclusive. In such situation we suggest the application of the following workflow to determine which normalization method is optimal for a specific data set: (i) normalize the data using considered methods, (ii) calculate the “bias” and “variance” and rank the methods based on these values, (iii) after each normalization perform differential analysis and determine DEG lists found by each normalization method, (iv) select a subset of genes that can serve as positive and negative controls to investigate the sensitivity and specificity of normalization methods and rank the methods based on these criteria, (v) calculate the percentage of the mean of the prediction errors obtained using chosen classifiers for DEGs found by each normalization method and rank them, (vi) draw Venn diagrams or balloon plots based on the number of differentially expressed genes and rank the methods based on the number of common DEG values, and (vii) based on the summary of ranks choose the most appropriate normalization method of the investigated data set. We notice here that the normalization method can influence the expression results, leading to erroneous DE analysis; therefore it is very important to put effort at this stage of the analysis. Finally, we wanted to draw attention that our paper does not indicate clearly which normalization method is the best, but it adds a new look at how to choose the normalization for RNA-Seq data analysis to avoid erroneous DE analysis. It can be applied not only to methods concerning sequencing depth but the proposed algorithm is also suitable to compare normalizations that take into account other sources of unwanted variation.

## 5. Conclusions

In the study, new coefficients such as bias and variance were proposed as objective criteria for a comparison of normalization methods. In conclusion, our results suggest that, depending on the RNA-seq data structure and the applied method, the influence of normalization differs. However, the presented criteria, in particular bias and variance, can support the choice of normalization method optimal for a specific data set.

## Supplementary Material

In Supplementary Materials we provide additional details concerning data sets used in the presented research. Furthermore, we provide here additional results not included in the main text for comparison of normalization methods, that include other sources of variation such as GC content and batch correction.

## Figures and Tables

**Table 1:** Summary of data set information.

Data set	Number of samples	Number of genes	Number of genes (after filtering)	Min of average abundance of gene from all genes	Max of average abundance of gene from all genes	Number of HK (housekeeping genes)
Cheung	41	52 580	12 410	0.024	90180	124
Bodymap	16	52 580	13 131	0	934100	131
AML	27	57 736	12 749	50.04	482400	127

**Table 2:** Classifiers used in the calculations, function in R, and the name and the version of R package.

Classification method	Function in R	R package	Version
Naive Bayes	NaiveBayesI	MLInterfaces	1.40.0
Neural network	nnetI	MLInterfaces	1.40.0
*k*–nearest neighbour	knnI	MLInterfaces	1.40.0
Support vector machines	svmI	MLInterfaces	1.40.0
Random forest	randomForestI	MLInterfaces	1.40.0

**Table 3:** Rank of the bias values obtained using five normalization methods and RD for the normalization of the three tested data sets.

Normalization method	Cheung data set rank (bias value)	Bodymap data set rank (bias value)	AML data set rank (bias value)
TMM	3.5 (0.890)	4 (1.123)	6 (0.587)
UQ	3.5 (0.890)	5 (1.139)	4 (0.564)
DES	1 (0.885)	2 (1.102)	1 (0.512)
EBS	2 (0.887)	3 (1.111)	3 (0.532)
PS	5 (0.893)	1 (1.098)	2 (0.523)
RD	6 (0.908)	6 (1.159)	5 (0.581)

**Table 4:** Rank of the variance values obtained using the five normalization methods and RD for the normalization of the three tested data sets.

Normalization method	Cheung data set rank (variance value)	Bodymap data set rank (variance value)	AML data set rank (variance value)
TMM	4 (0.779)	4 (1.305)	6 (0.364)
UQ	3 (0.778)	5 (1.330)	4 (0.335)
DES	1 (0.768)	2 (1.270)	1 (0.283)
EBS	2 (0.771)	3 (1.283)	3 (0.300)
PS	5 (0.782)	1 (1.268)	2 (0.291)
RD	6 (0.812)	6 (1.390)	5 (0.355)

**Table 5:** Sensitivity and specificity of the studied five normalization methods and RD applied to the AML data set.

Methods	TMM	UQ	DES	EBS	PS	RD
Sensitivity (%)	11.4	20.5	18.2	45.5	40.9	18.2
Rank of sensitivity	6	3	4.5	1	2	4.5
Specificity (%)	97.7	84.1	97.7	52.3	65.9	97.7
Rank of specificity	2	4	2	6	5	2

**Table 6:** Performances of the normalization methods with informative genes based on five classifiers and LOOCV applied to the Cheung, Bodymap, and AML data sets. The first number in each cell denotes the percentage of the average of five prediction errors from five different classification methods. The second number in each cell that is in brackets is the percentage of the median of the five prediction errors.

	TMM	UQ	DES	EBS	PS	RD
Cheung	11.1	10.7	4.5	4.4	3.9	11.6
	(9.8)	(7.3)	(0.4)	(0.0)	(0.0)	(12.2)

Bodymap	16.2	16.2	16.0	15.8	16.0	16.2
	(18.8)	(18.8)	(17.7)	(16.7)	(17.7)	(18.8)

AML	8.1	8.0	7.7	7.4	7.3	8.3
	(7.4)	(7.4)	(7.4)	(7.4)	(7.4)	(7.4)

**Table 7:** Summary of comparison results for the five normalization methods under consideration. The final rank is based on the bias and variance values, the prediction errors, sensitivity, specificity values, and the number of common DEGs for AML data.

	TMM	UQ	DES	EBS	PS
Bias	5.0	4.0	1.0	3.0	2.0
Variance	5.0	4.0	1.0	3.0	2.0
Sensitivity	5.0	3.0	4.0	1.0	2.0
Specificity	1.5	3.0	1.5	5.0	4.0
Prediction errors	5.0	4.0	3.0	2.0	1.0
Common DEGs	5.0	2.0	1.0	4.0	3.0
