# Supplementary material for: The Impact of Normalization Methods on RNA-Seq Data Analysis
Source: Biomed Res Int. 2015 Jun 15;2015:621690. doi: 10.1155/2015/621690 (PMC4484837; doi:10.1155/2015/621690)
Supplement: Supplementary file 1 — In Supplementary Materials we provide additional details concerning data sets used in the presented research. Furthermore, we provide here additional results not included in the main text for comparison of normalization methods, that include other sources of variation such as GC content and batch correction. [file 621690.f1.pdf]

Supplementary Materials

for "The impact of normalization methods on RNA-seq data analysis "

Authors: Zyprych-Walczak J.[1], Szabelska A.[1], Handschuh L.[2,3], Górczak K.[1], Klamecka K.[1], Figlerowicz M.[2] and Siatkowski I.[1]

[1]Department of Mathematical and Statistical Methods, Poznan University of Life Sciences, 60-637 Poznan, Poland; [2]Institute of Bioorganic Chemistry, Polish Academy of Sciences, 61-704 Poznan, Poland; [3]Department of Hematology and Bone Marrow Transplantation, Poznan University of Medical Sciences, 60-569 Poznan, Poland.

Corresponding author : Joanna Zyprych-Walczak, Department of Mathematical and Statistical Methods, Poznan University of Life Sciences, Wojska Polskiego 48, 60-637 Poznan, Poland. Tel.: +48 61-848-7550; E-mail: zjoanna@up.poznan.pl.

Joanna Zyprych-Walczak is a biostatistician at Poznan University of Life Sciences in Poland at the Department of Mathematical and Statistical Methods. Her projects focus on microarray and RNA-seq data analysis. She deals mainly with methods for determining differentially expressed genes based on gene expression values.

Alicja Szabelska is a biostatistician at Poznan University of Life Sciences in Poland at the Department of Mathematical and Statistical Methods. Her research interests focus on the development of statistical methodology for the analysis of high-throughput 'omics data.

Luiza Handschuh heads the Laboratory of Microarrays and Deep Sequencing of the IBCH PAS Center of Genomics, employed also at the Department of Hematology and Bone Marrow Transplantation, Poznan University of Medical Sciences. As an expert in microarrays and NGS technologies, she participates in numerous projects held by IBCH PAS and other units cooperating with the Institute.

Katarzyna Górczak is a Ph.D. student in biostatistics at Poznan University of Life Sciences in Poland at the Department of Mathematical and Statistical Methods. Her projects focus on RNA-seq data analysis.

Katarzyna Klamecka was a Ph.D. student in biostatistics at Poznan University of Life Sciences in Poland at the Department of Mathematical and Statistical Methods. Her projects focused on RNA-seq data analysis.

Marek Figlerowicz holds degrees in chemistry and biology, and has a wide experience in carrying out national and international projects that apply the tools of genomics and proteomics. Professor Figlerowicz heads the European Center of Bioinformatics and Genomics (ECB&G) and the Department of Molecular and Systems Biology at the IBCH PAS, and since 2011 he has been the Director of the IBCH PAS.

Idzi Siatkowski is a Professor at the Department of Mathematical and Statistical Methods at Poznan University of Life Sciences in Poland. He is the coordinator of the NGS data analysis group of the Bioinformatics Institute.

Contents

1 Supplementary Figures 3

2 Supplementary Tables 10

1 Supplementary figures

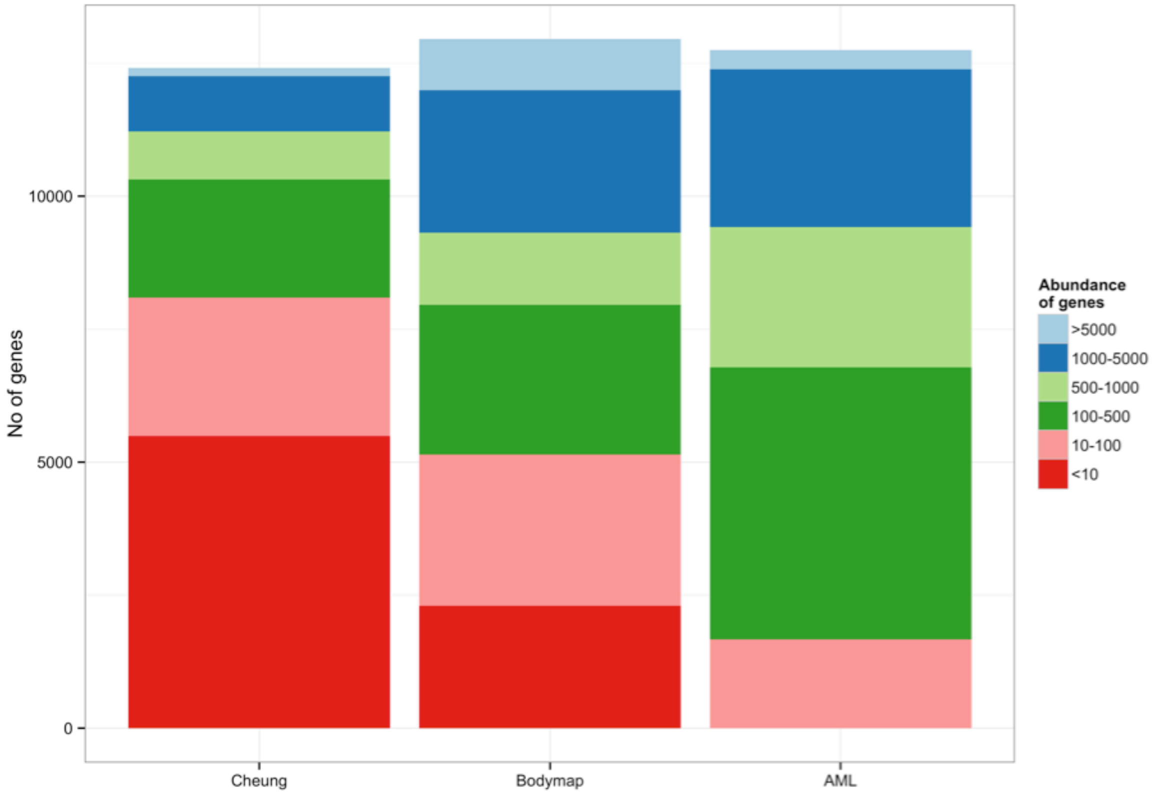

Figure S1. Barplots presenting the number and composition of genes analyzed for each data set. Distinguished 6 levels of count of mean abundances are presented in colors.

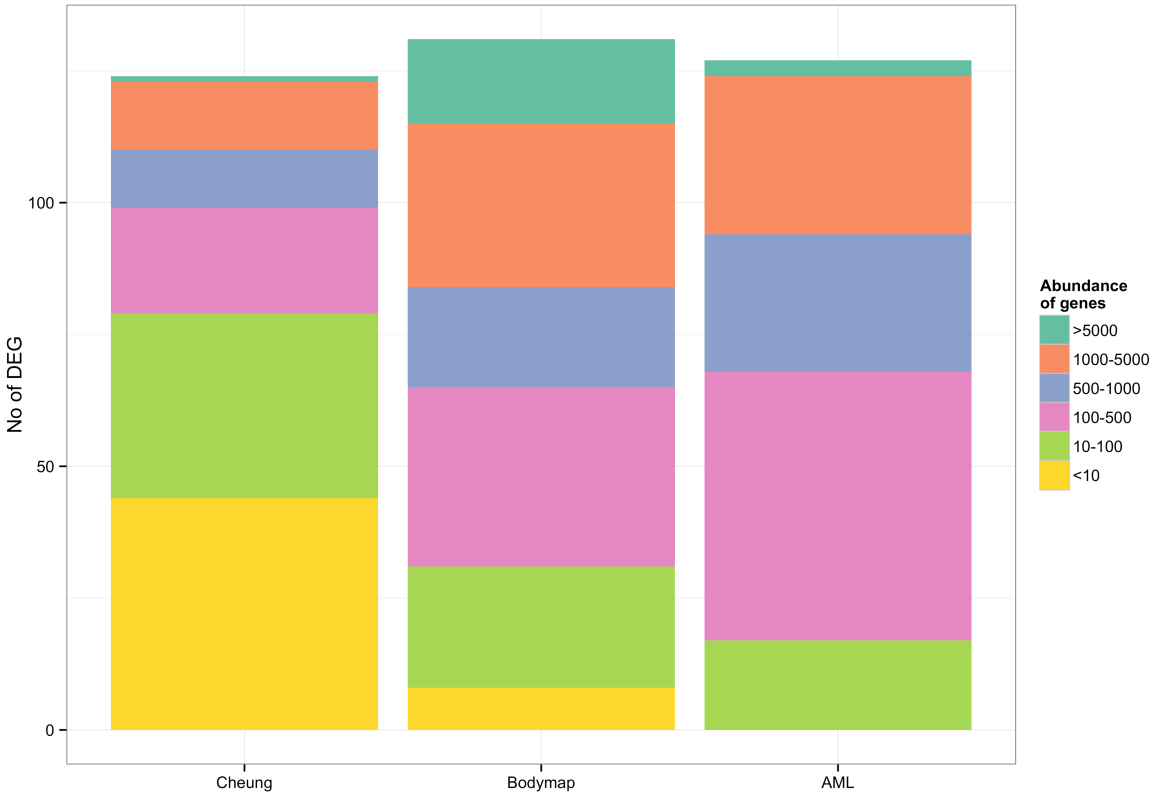

Figure S2. Barplots presenting the number and composition of housekeeping genes for each data set. Distinguished 6 levels of count of mean abundances are presented in colors.

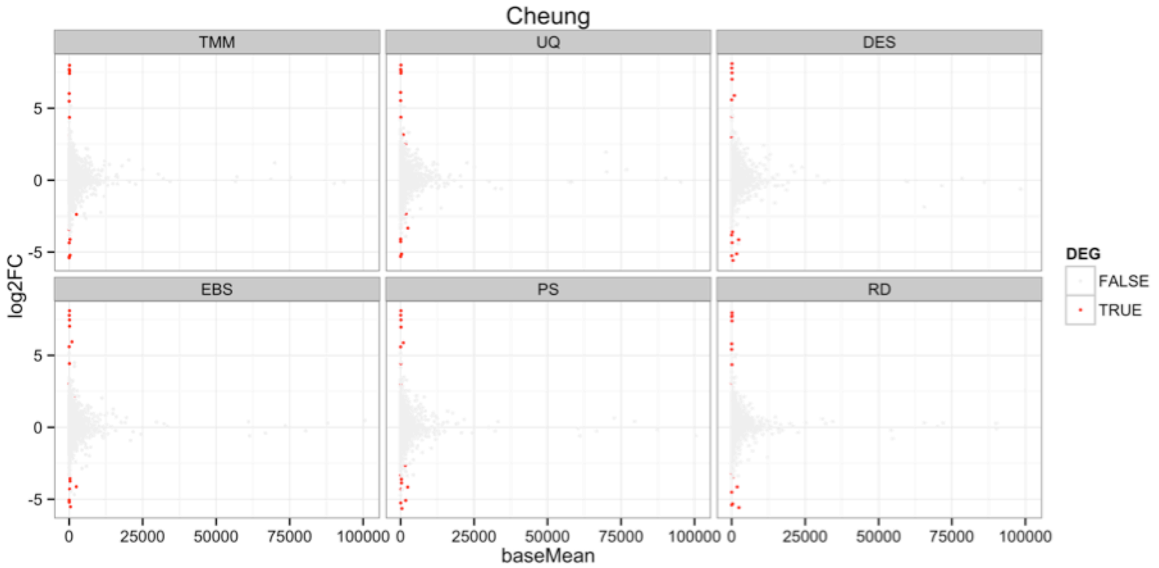

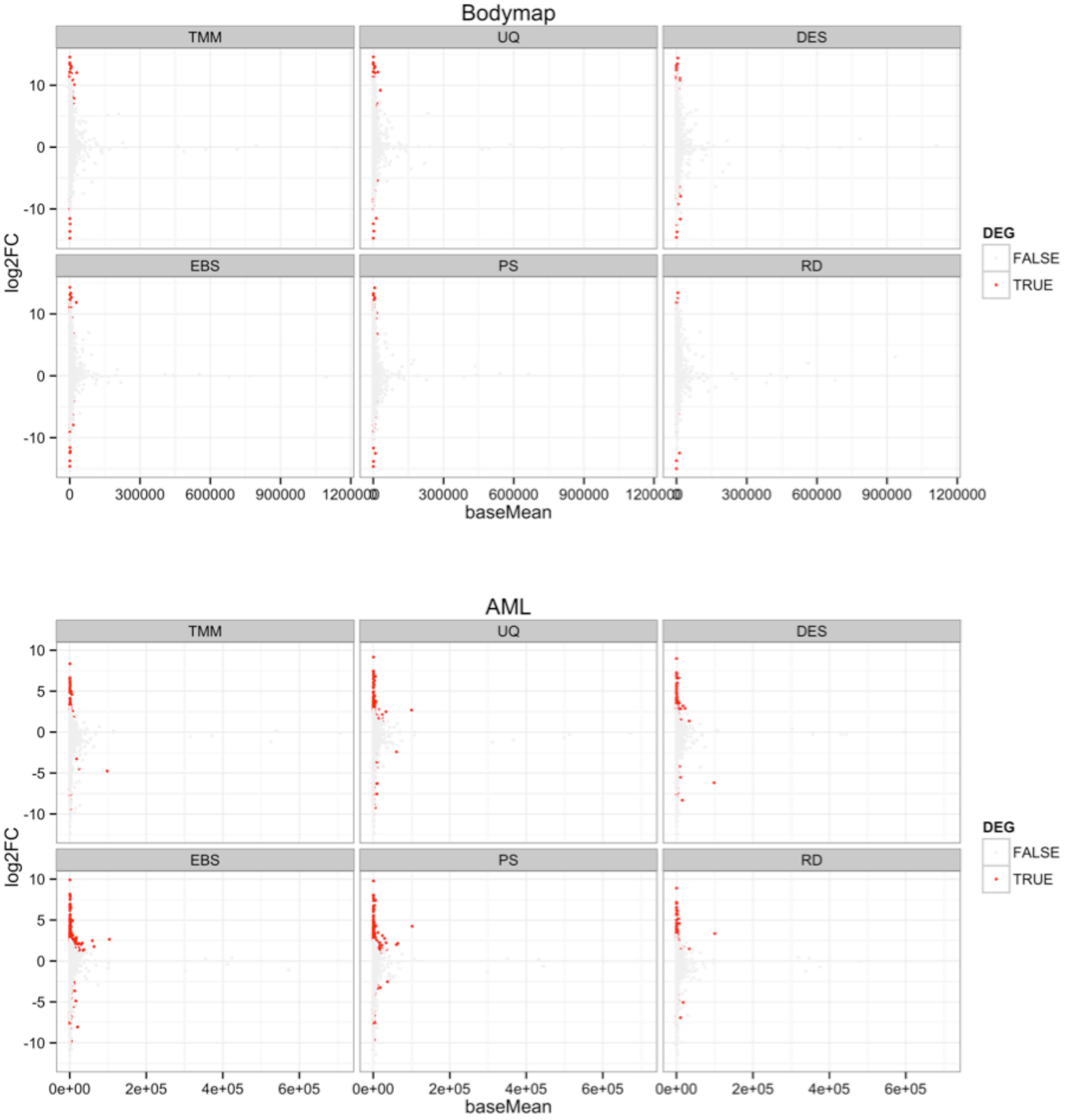

Figure S3. MAplots presenting the raw data (RD) and data after 5 methods of normalization, in the case of Cheung, Bodymap and AML data sets. Each dot represents one gene. Red dots represent DEGs.

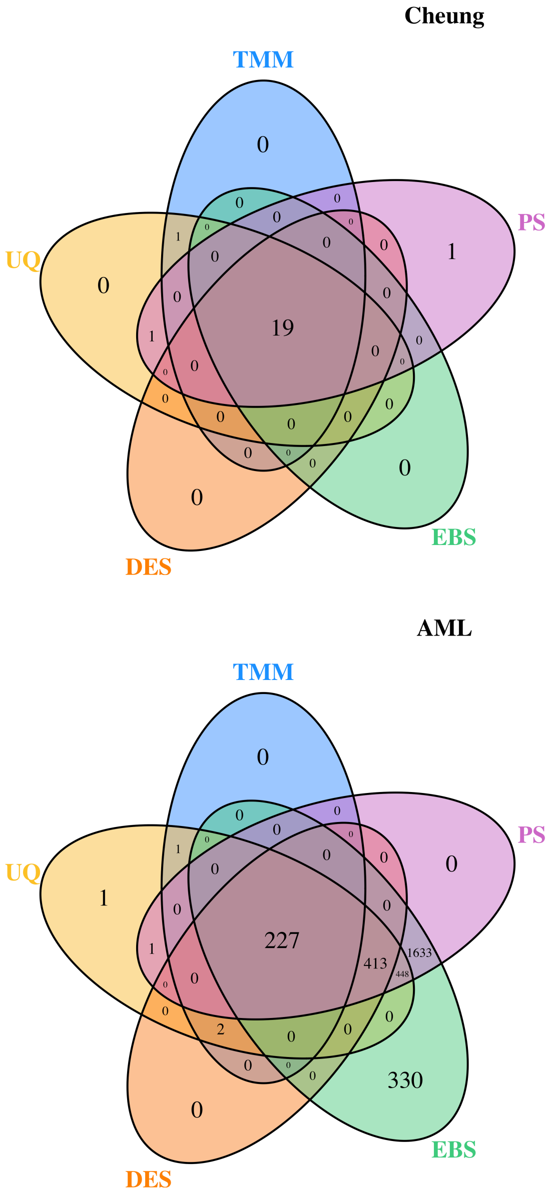

Figure S4. Venn diagrams for five normalization methods based on Cheung, Bodymap and AML data sets.

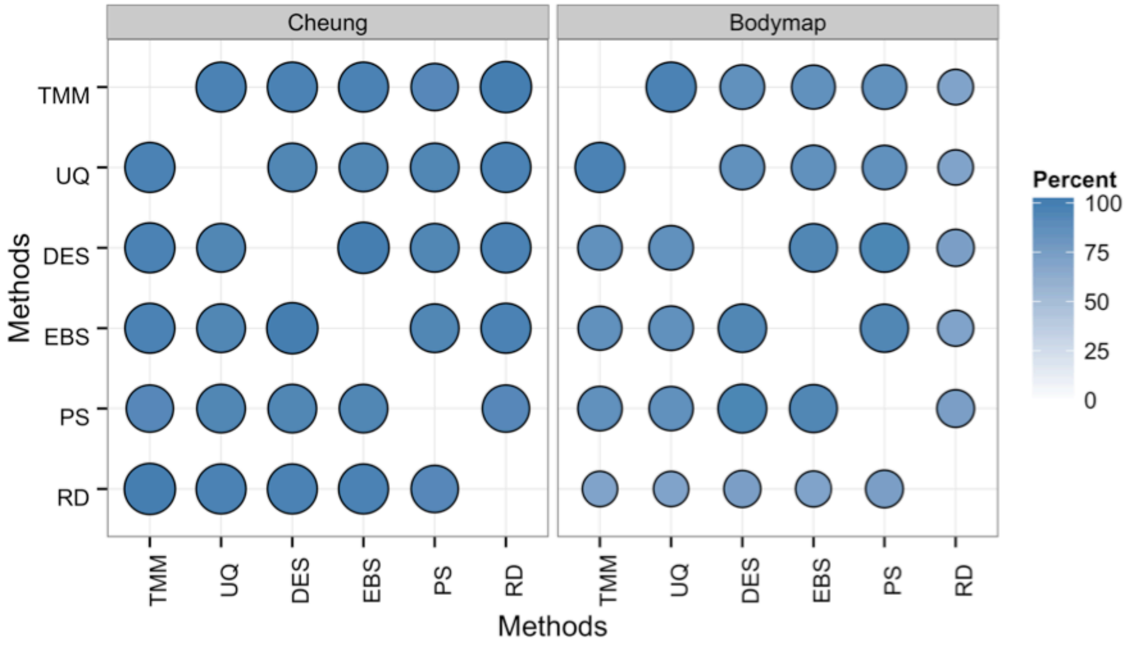

Figure S5. Common DEGs across each pair of normalization methods for Cheung and Bodymap data sets. The sizes and shadowing of circles represent the percentage value of common genes.

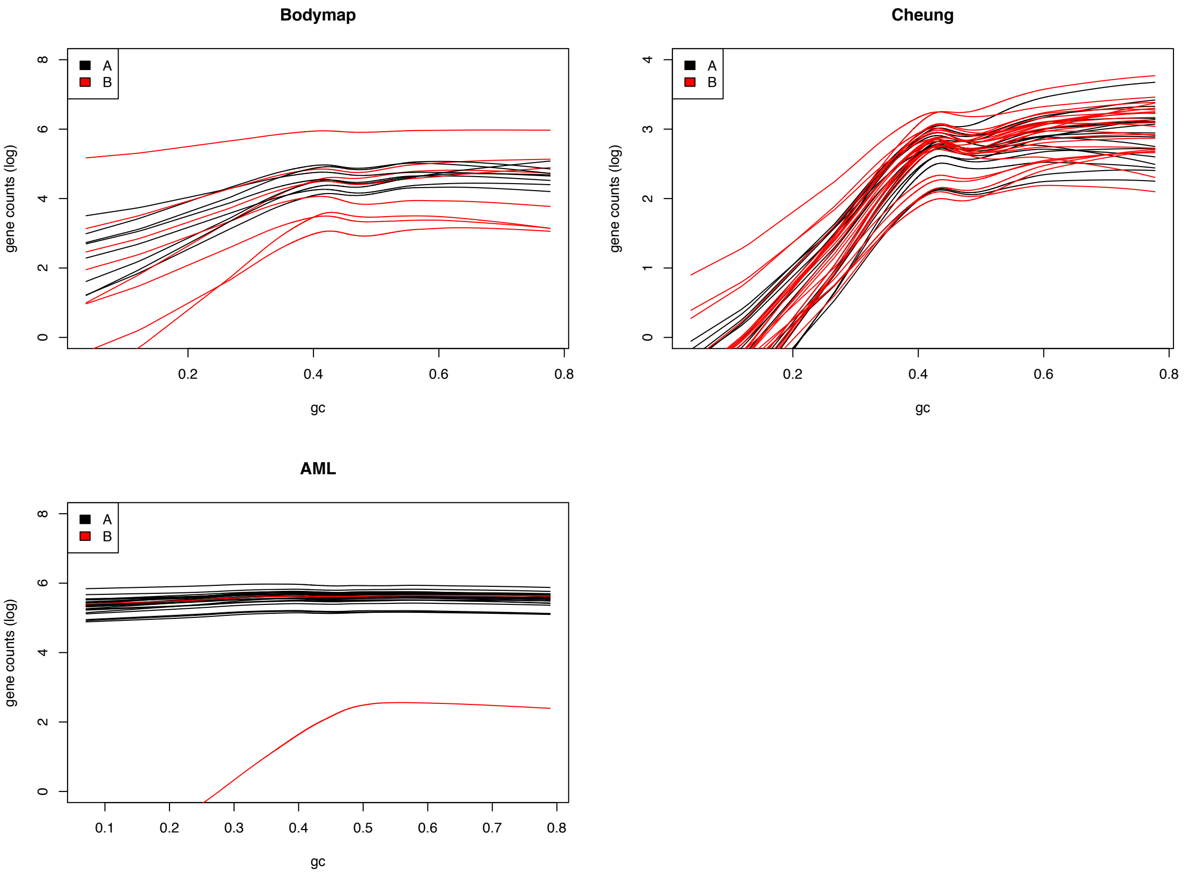

Figure S6. Log-transformed counts versus GC-content for each sample in the Cheung, Bodymap and AML data. Replicates in each condition are represented by the same color, as indicated in the legends.

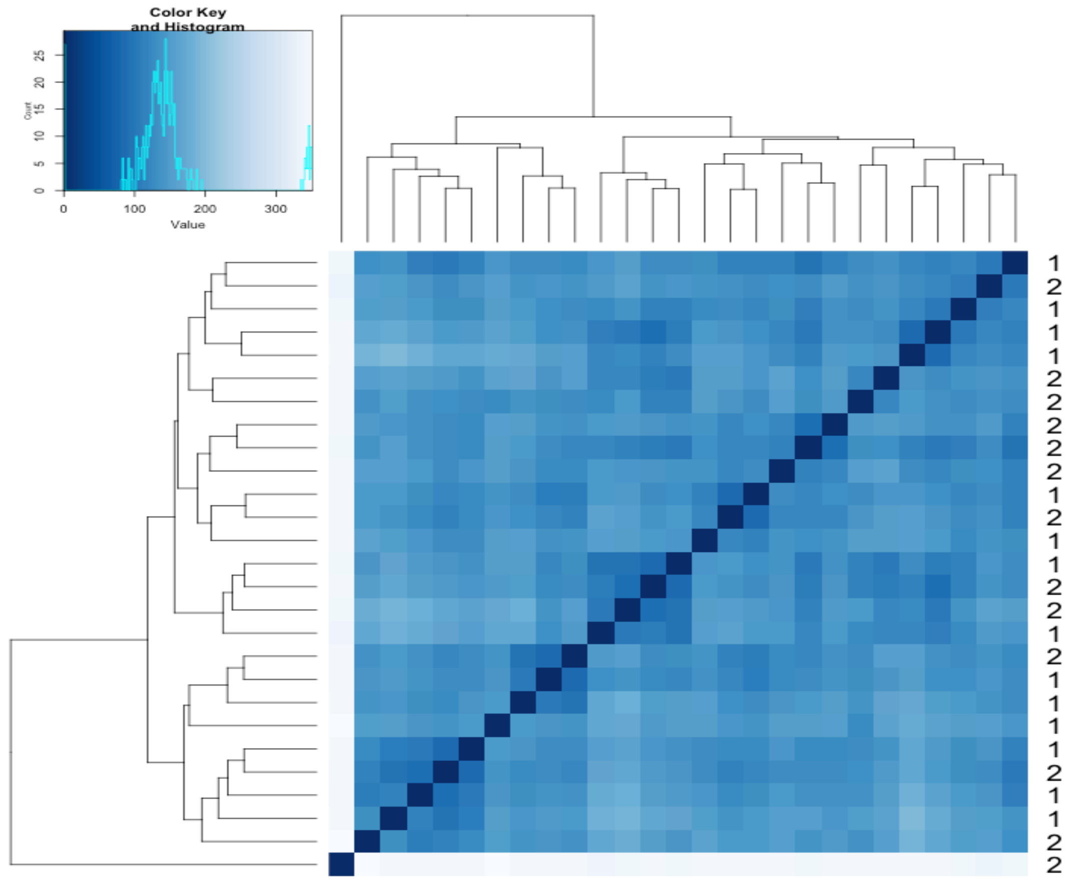

Figure S7. Heatmap of distances between samples based on log read counts for AML data set. The dendrogram was created based on hierarchical cluster analysis with complete method. The labels of rows indicate the number of batch connected with sampling date.

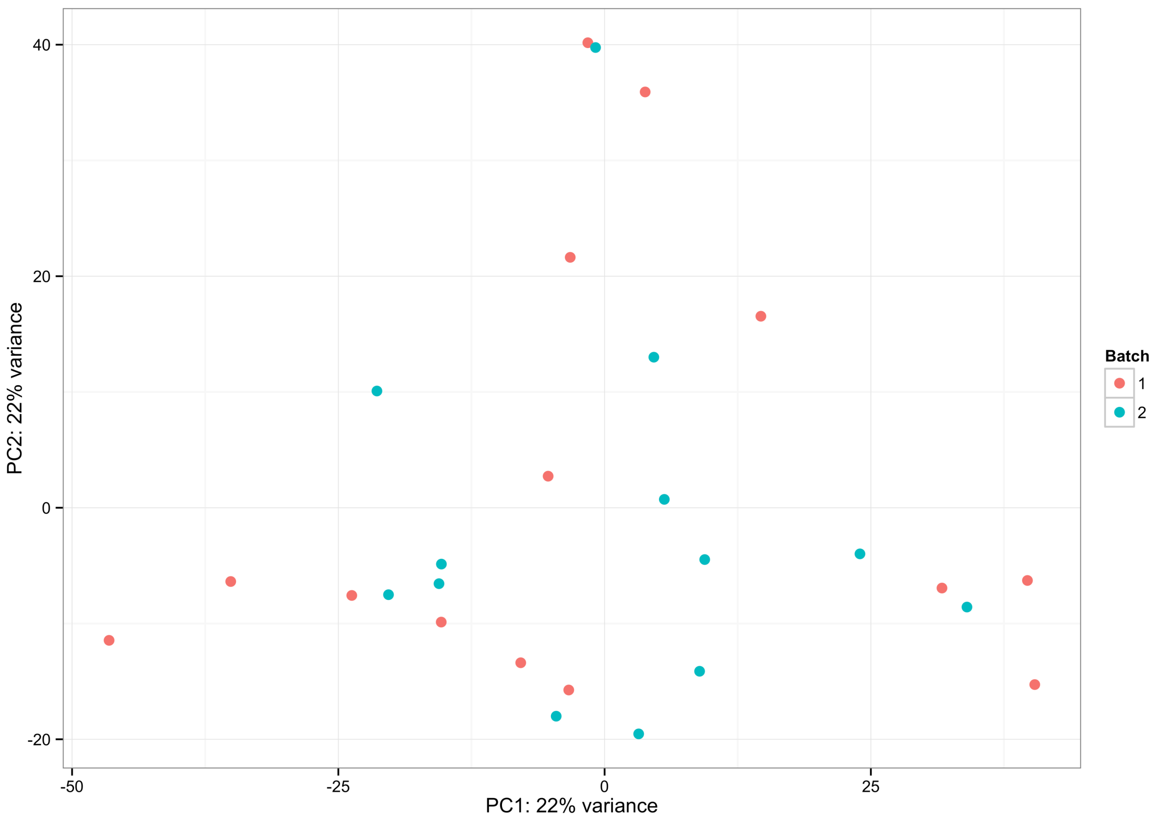

Figure S8. PCA (principal-components analysis) visualization of the first two principal components for the AML data set. The colors in the plot indicate the batch number that is connected with sampling date.

2 Supplementary tables

Table S1. The number of genes for particular mean of abundances for all genes in each datasets

Mean of abundance of genes	Number of genes in each dataset<br>Cheung	Bodymap	Leukemia
<10	5492	2300	0
10-100	2602	2845	1669
100-500	2220	2811	5114
500-1000	902	1357	2635
1000-5000	1041	2677	2965
>5000	152	963	365

Table S2. The number of housekeeping genes for particular mean of abundances for all housekeeping genes in each datasets.

Mean of abundance of HG	Number of HG genes in each dataset<br>Cheung	Bodymap	Leukemia
<10	44	9	0
10-100	33	24	15
100-500	21	34	46
500-1000	14	20	31
1000-5000	12	30	31
>5000	1	14	4

Table S3. The list of positive control genes and negative control genes.

negative control genes		positive control genes	
ACADVL	PSEN1	AGER	KRAS
ADSL	PSMB2	AURKA	MCL1
BTD	PSMB4	AURKB	MSLN
C1orf43	RAB7A	AURKC	MTHFD1
CANX	RAC2	BAALC	MUC1
CHMP2A	REEP5	BCL2	NPM1
CLU	RPL11	BIRC5	NRAS
EMC7	RPL19	BMI1	NSD1
FTL	RPL37A	CALML4	NUDCD1
G6PD	RPL5	CCNA1	PRAME
GPI	RPLP0	CCNB1	PRKCSH
H3F3A	RPLP1	CCNE1	PRTN3
HPRT1	RPS27A	CDC25C	RGS5
HSP90AA1	RPS29	DNAJA1	RPS23
LDHA	RPS3	DNAJC2	RPSA
MT2A	SNRPD3	FLT3	SAGE1
NONO	TCEA1	HBG2	SPAG9
PFKL	TMSB4X	HMMR	SSX2IP
PFKM	TUBA1A	HN1L	SYCP1

PFKP	VCP	HOXA9	TERT
PGAM1	VPS29	HRAS	USP33
PGK1	VPS72	ING3	WT1

Table S4. Summary of comparison results for the five normalization methods under consideration. The final rank is based on the bias and variance values, sensitivity, specificity values, the prediction errors and the number of common DEGs for AML data after additionally 'gc content – EDAseq' normalization.

Criteria	TMM	UQ	DES	EBS	PS
bias	5(0.818)	4(0.787)	1(0.748)	3(0.766)	2(0.754)
variance	5(0.689)	4(0.636)	1(0.587)	3(0.606)	2(0.592)
sensitivity	5(4.167)	3(20.83)	4(12.50)	1(37.50)	2(29.17)
specificity	1(96.55)	3(82.76)	2(93.10)	5(48.28)	4(58.62)
prediction errors	1(7.160)	4(9.506)	5(9.877)	3(8.889)	2(8.395)
common DEGs	5(48.75)	1(57.17)	2(57.08)	4(54.08)	3(56.50)
